# A Machine Learning Study on the Thermostability Prediction of (R)-*ω*-Selective Amine Transaminase from Aspergillus terreus

**DOI:** 10.1155/2021/2593748

**Published:** 2021-08-16

**Authors:** Li-li Jia, Ting-ting Sun, Yan Wang, Yu Shen

**Affiliations:** School of Science, School of Big Data, Zhejiang University of Science and Technology, Hangzhou 310008, China

## Abstract

Artificial intelligence technologies such as machine learning have been applied to protein engineering, with unique advantages in protein structure, function prediction, catalytic activity, and other issues in recent years. Screening better mutants is still a bottleneck in protein engineering. In this paper, a new sequence-activity relationship method was analyzed for its application in improving the thermal stability of Aspergillus terreus (R)-*ω*-selective amine transaminase. The experimental data from 6 single-point mutated enzymes were used as a learning dataset to build models and predict the thermostability of 2^6^ mutants. Based on digital signal processing (DSP), this method digitized the amino acid sequence of proteins by fast Fourier transform (FFT) and then established the best model applying partial least squares regression (PLSR) to screen out all possible mutants, especially those with high performance. In protein engineering, the innovative sequence activity relationship (ISAR) method can make a reasonable prediction using limited experimental data and significantly reduce the experimental cost. The half-life (*T*_1/2_) of (R)-*ω*-transaminase was fitted with the amino acid sequence by the ISAR algorithm, resulting in an *R*^2^ of 0.8929 and a cvRMSE of 4.89. At the same time, the mutants with higher *T*_1/2_ than the existing ones were predicted, laying the groundwork for better (R)-*ω*-transaminase in the later stage. The ISAR algorithm is expected to provide a new technique for protein evolution and screening.

## 1. Introduction

The natural enzyme, as a biocatalyst, is active under biological or natural conditions, but its activity is so poor in the actual production system that it cannot be well applied. In addition, the disadvantages such as poor stereo/regioselectivity, low catalytic efficiency, and poor stability hinder the wide application of biocatalysts. Enzyme-directed evolution is indispensable in biocatalysis, biomedicine, and biotechnology. In organic chemistry and biotechnology, the directed evolution of stereoselective, regioselective, and chemoselective enzymes as catalysts provides vast resources for various transformations in organic synthesis and biotechnology [1–3]. The rapid development of computational biology has enabled many software and databases to analyze large amounts of sequence information rapidly. The semirational design is based on sequence alignment, commonly using the HotSpot Wizard sever [4], 3DM database [5], probe [6], etc., as tools. The QM/MM combination method based on classical mechanics and quantum mechanics is considered one of the most reliable computational simulation methods to study the mechanism of enzyme catalysis [7]. Extracting possible target sites from the database in combination with other rational analysis methods can improve the stability of the enzyme quickly and effectively [8]. By simulating the natural evolution, the target genes were mutated, expressed, and screened many times to finally obtain the proteins with improved properties or new functions [9].

In protein engineering, the initial library was generated and screened by experimenters. In directed evolution, diversified mutant libraries were caused by error-prone polymerase chain reaction, saturation mutation, and DNA shuffling. In a 1997 study on the principle of stereoselectivity in directed evolution, it was reported that four cycles of epPCR were used to increase the stereoselectivity of lipase by 10 times [10, 11]. Besides, saturation mutagenesis can be combined with epPCR, DNA shuffling, and other techniques to rapidly accumulate sense mutations and obtain the best combination of enzyme genes. For example, the Reetz team pioneered the comprehensive use of these three traditional techniques to improve the enantioselectivity of lipase [10, 12]. Then, a combined active site saturation test (CAST) based on the information of amino acid sequence and/or spatial structure, proposed by the Reetz team, was proved to be effective [13]. In addition, with the help of computer simulation, the active center of enzyme catalysis was directly related to the substrate. Later, iterative saturation mutation (ISM) [14–16] further improved stereoselectivity as the mutant library generated by CAST could not meet all the requirements of biochemical properties. The CAST/ISM methods were based on semirational strategies and widely used to modify enzyme parameters such as stereoselectivity/regioselectivity, substrate spectrum, and catalytic efficiency [17, 18].

Machine learning is a new way to improve efficiency by effectively designing or enzyme-directed evolution to produce fewer mutant libraries and high-demand mutant libraries [19, 20]. Machine learning is an extension of mathematical statistics and computer science, including statistical models and many algorithms for improving computer programs. Machine learning algorithm in its early development was used by Muggleton et al. in 1992 to predict secondary structure in protein science [21] and in 2008 was used in a physicochemical feature-based classification of amino acid mutations [22]. Later, a new version of machine learning was able to predict structure, folding, binding, and even catalytic activity to deal with cumulative information about mutants and their properties [23–29]. Experimental data serving as a training set for these algorithms can help predict new and improved variants, thus contributing to protein engineering experiments based on site-specific mutagenesis or enzyme-directed evolution [30, 31].

Transaminase is a biocatalyst with high stereoselectivity and mild reaction conditions, which can be used to prepare chiral intermediates by asymmetric synthesis and kinetic resolution. With the expanding market of chiral drugs, the industrial production of transaminase has shown great potential and broad application prospects. However, most transaminases have suffered from poor thermostability in nature, which severely limits their application in industrial production. Therefore, improving the thermostability of enzymes is both a challenge for transaminase protein engineering and a pressing issue for transaminase application in industrial production. *ω*-Transaminase (*ω*-TA) is a natural biocatalyst that can directly synthesize enantiomer chiral amines. Compared with (S)-*ω*-TA, (R)-*ω*-TA [32] was less studied. Despite the poor thermostability of (R)-*ω*-TA, its demand increased with the march of chiral amines. The improvement of the thermostability of (R)-*ω*-TA with potential application value is expected to benefit the preparation of chiral amines. (R)-*ω*-transaminase from Aspergillus terreus was used to obtain a small mutant library by rational design. The digital signal processing (DSP) was carried out according to the existing data. Then, the half-life (*T*_1/2_) of the wild-type enzyme and the mutant enzyme was modeled and predicted to obtain the mutant enzyme that could fit the existing data and improve the thermostability. *T*_1/2_ denotes the time for the residual activity of (R)-*ω*-transaminase to be reduced to 50% of its original activity at 40°C [33].

In this study, a mutant screening method called ISAR (innovative sequence activity relationship) was used to deal with the sequence-function correlation of biological macromolecules in many aspects, including the physical and chemical properties of amino acids, DSP. Besides, partial least square regression was used to reveal these sequence-function correlations [34]. At present, there are very limited articles on the use of the machine learning method to study (R)-*ω*-TA. In this study, the python tool was employed to write the ISAR algorithm, which combines machine learning with protein engineering. It is expected to provide new ideas for later research effectively.

## 2. ISAR Method

**Figure 1:**
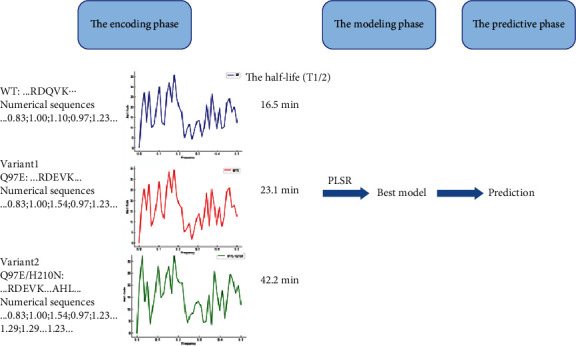
The flow chart of ISAR methodology.

ISAR comprises the encoding phase, the modeling phase, and the predictive phase, which are consistent with the fundamental processes of mathematical statistics (Figure 1). As shown in Figure 1, the sequences of WT and two variants were encoded to numerical sequences based on an index of AAindex databases. The numerical sequences were transformed into protein spectra by FFT. Then, a regression model was constructed with protein spectra and *T*_1/2_ being learning datasets. Finally, *T*_1/2_ of new variants were predicted by the model.

In the first phase, the encoding phase, all protein sequences were required to be encoded into digital sequences by the better index of the AAindex database. So far, the AAindex database comprises 566 amino acid indexes, which represent the biochemical and physicochemical properties of 20 standard amino acids. The correlations between these indexes were listed. The amino acid sequence of protease was digitized using DSP technology. DSP is an analytical program that decomposes and processes signals to display embedded data [35]. The data signals processed by DSP can be discrete or continuous, such as DNA, RNA, and protein residues, representing the biological information of these biological macromolecules. Protein-protein interactions were analyzed by the DSP technique [36], which also converted protein properties into protein spectral form. In protein engineering, the Fourier transform of DSP was often used for protein and DNA comparison [37], characterization of protein families, pattern recognition [38–40], etc.

The DSP uses digital operation to realize signal transformation, filtering, detection, estimation, modulation and demodulation, fast algorithm, and so on. FFT was one of them. First, a protein sequence was encoded based on a better index of the AAindex [41, 42] database (https://www.genome.jp/aaindex/). Then, according to the energy-frequency representation (equation (1)), the coding sequence was converted into a protein spectrum by FFT.

(1) $$\begin{array}{c} S\left(k\right)=\overset{N-1}{\underset{n=0}{\sum }}s\left(n\right)e^{\left(-2i\pi k_{N}^{n}\right)}, \end{array}$$

where *s* is the input signal of length *N* (the coding sequence), *S* is the output spectrum (the complex number), *k* is the frequency in the spectrum, *n* is the position in the input signal, and *i* is the complex number that *i*^2^ = −1.

After numerical encoding, data standardization is an important stage of data preprocessing. Then, zeros were filled at the end of the digital sequence. FFT was used to convert the digital sequence into a protein spectrum. Zero filling accelerates the FFT algorithm [43] and allows for protein profiles of the same length in the case of different sequence lengths.

In the second phase, the modeling phase, the model was established with the partial least squares method for protein spectra from existing mutants and the activity of experimental data. The model verification for a small amount of data usually uses leave-one-out cross-validation (LOOCV) [30]. By LOOCV, the dataset was divided into *k* parts. One copy was used in the training of the model, and the remaining one was used in the test set. After all the cross-validation rounds, the overall performance of each test set was calculated. In this study, cross-validation was used to prevent overfitting and optimize some parameters (*R*^2^, cvRMSE, and other parameters). The minimum root-mean-square error (cvRMSE) and the determination coefficient (*R*^2^) in range verification were used as the best AAindex to optimize the model. The prediction of the model depended on the *R*^2^ value. cvRMSE was able to build and select the best model and prevent overfitting. The *R*^2^ and cvRMSE were interpreted as the definitions shown in

(2) $$\begin{array}{c} R^{2}=\frac{{\left(\sum_{\text{i}=1}^{S}\left(y_{i}-\bar{y}\right)\left({\overset{\wedge }{y}}_{i}-\overset{\wedge }{\bar{y}}\right)\right)}^{2}}{\sum_{i=1}^{S}{\left(y_{i}-\bar{y}\right)}^{2}\sum_{i=1}^{S}{\left({\overset{\wedge }{y}}_{i}-\overset{\wedge }{\bar{y}}\right)}^{2}},\\ \text{cvRMSE}=\sqrt{\overset{S}{\underset{i=1}{\sum }}\frac{{\left(y_{i}-{\overset{\wedge }{y}}_{i}\right)}^{2}}{S}}, \end{array}$$

where *y*_*i*_ is the experimental activity of the *i*th sequence, ${\hat{y}}_{i}$ is the predicted activity of the *i*th sequence by ISAR, $\bar{y}$ is the average of the experimental activity, and *S* is the number of sequences.

In the last phase, the predictive phase, partial least squares regression (PLSR) modeling was carried out according to all spectra from experimenting data and certain activity. Since each location of the amino acid sequence may or may not mutate, the *n* single point mutations will produce 2^*n*^ of all possible mutants. The model obtained in the modeling phase of 2^*n*^ mutants was well predicted. Variants with higher performance could be obtained. Therefore, the limited experimental data was supposed to be used as a training set for these algorithms to predict new and improved variants, thus assisting the experimental work of protein engineering based on site-specific mutations or enzyme-directed evolution [44].

## 3. Discussion

### 3.1. Experimental Dataset

The experimental data was from Xie's team [33]. Stabilized variants of the (R)-*ω*-transaminase from Aspergillus terreus were constructed by consensus mutagenesis. *T*_1/2_ was defined as the time when the residual activity of (R)-*ω*-transaminase was reduced to 50% of its original activity at 40°C. Six-point mutations and combinations of mutations were under the same chemical reaction. There was a little error in the *T*_1/2_ reaction in the same environment. In this paper, the average value obtained from many experiments was used.

The improved thermal stability of (R)-*ω*-TA, a transaminase with 325 amino acid residues, is of great significance to the result of half-life (T_1/2_). In the present study, zeros were added to the digital sequence from 325 to 512 (2^9^) to speed up the fast Fourier transform (FFT) algorithm.

A relatively good mutant was selected from all the possible six-point (I77L, Q97E, H210N, N245D, G292D, and I295V) combinations of mutants (64). Using the ISAR approach, 13 limited pieces of data were obtained from the experimental group for 6 single-point mutations and combinations of mutations. Under certain conditions, the *T*_1/2_ values of these mutants were used to measure the thermostability of (R)-*ω*-TA. The *T*_1/2_ of wild-type (WT) (R)-*ω*-TA was 6.9. The best measurement mutant described in the study was P7, which included all six single-point mutants and combined mutants in the sequence, with a *T*_1/2_ value of 42.2. In the case of only 13 limited mutants, the model can predict the half-life of Aspergillus terreus (R)-*ω*-TA of 64 mutants by ISAR, so we can get better new mutants than experimenting with data whose *T*_1/2_ was higher than 42.2. The best mutation available for existing data was P7 (42.2). Several mutants larger than 42.2 were obtained by the ISAR method.

### 3.2. Selection of the Best Modeling Index

**Figure 2:**
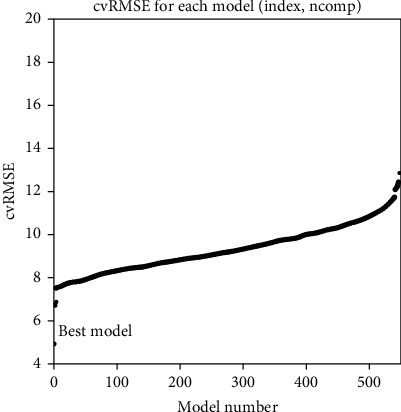
CvRMSE for 549 models. Based on the construction and testing of 549 models, the evolution of root mean square error of cross-validation of *T*_1/2_ was predicted, where the horizontal axis is 549 models and the vertical axis is the cvRMSE value of each model. The best model is the leftmost point, where the value of cvRMSE is 4.89.

The index of the AAindex database digitized the amino acid sequence of (R)-*ω*-TA, where the value of each amino acid represented the corresponding biochemical characteristics. As the database was updated, it currently held 566 indexes. 17 groups with imperfect or missing values in the AAindex database were discarded, leaving 549 sets of indexes left for choosing the best one to use. Finally, 549 models were constructed corresponding to the remaining 549 indexes. The AAindex entries corresponding to the better cvRMSE and *R*^2^ models with the best performance were selected for the numerical coding of PLSR. Each coding metric was evaluated by ISAR to get the best index for building the model. Protein sequences were encoded and converted into a protein spectrum by FFT using each index. Using training data, ISAR built a prediction model for each indicator through PLSR. Based on the modeling of protein amino acid sequence, the number of principal components obtained determines the pros and cons of the PLSR algorithm. Different components and the LOOCV method made better performance parameters (*R*^2^ and cvRMSE) of each model (Figure 2).

Furthermore, the best index was NAGK730101, among the top 10 best indexes (Table 1). For the best index, the *R*^2^ and cvRMSE values were, respectively, 0.8929 and 4.89.

### 3.3. Modeling and Activity Prediction

**Figure 3:**
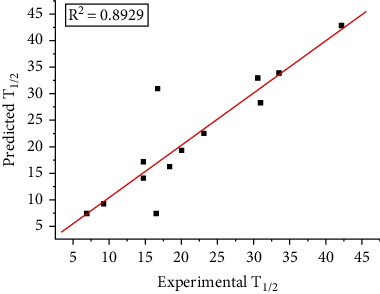
Prediction of *T*_1/2_ about (R)-*ω*-TA and mutants by LOOCV, *R*^2^ = 0.8929.

After evaluating multiple indicators in the AAindex database, the optimal index, NAGK730101, was selected based on the minimum cvRMSE and better *R*^2^. Moreover, the ISAR method was used to model and predict the (R)-*ω*-TA. The experimental data of (R)-*ω*-TA WT and mutants were digitized and converted into a protein spectrum for PLSR. Different models were obtained by various indexes, other parameters, different *R*^2^, and cvRMSE. According to the robustness of the model, the model received from each training set (12 pieces of data) was selected to predict. Then, the 13 predicted values obtained by 13 models were compared with the experimental values, as shown in Table 2. ISAR used two steps to calculate the values of model performance parameters. The first step was standard cross-validation in machine learning; the second, modeling using all data sets in the learning step. Based on the information of 13 data mutation points, a relatively good *R*^2^ value of pattern LOCCV (0.8929) was obtained (Figure 3).

**Figure 4:**
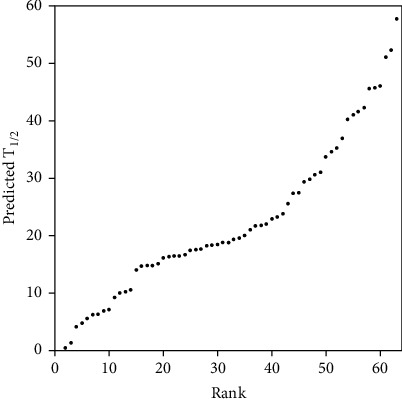
Ranking of the *T*_1/2_ for the 64 possible variants of transaminase with ISAR where the horizontal is 64 possible ranked variants and the vertical axis is the predictive value of *T*_1/2_.

Six sites were either mutated or not mutated by ISAR's machine learning algorithm, and 2^6^ (64) combinations were obtained. Then, the *T*_1/2_ values of all possible combinations (64 possible variants) of these limited variants were predicted by the best model.  Figure 4 shows the prediction of all possible mutants in the training set. At the same time, it was found that this model can identify new mutants with better thermal stability than P7. The thermal stability (*T*_1/2_) of (R)-*ω*-TA was predicted by the best model and ranked according to the *T*_1/2_ value of the mutant (Figure 4). After ranking the 64 possible mutant predictions, excellent mutants with a half-life greater than the existing experimental data were obtained. The best mutation available for existing data was P7 (42.2). Several mutants larger than 42.2 were yielded by the ISAR method.

64 possible mutant predictions were received, and the remaining 6 mutants' *T*_1/2_ (Table 3) were listed by ISAR. The highest mutant was P13 (Q97E_H210N_G292D_I295V), and the half-life value was 57.81. Then, the mutant's *T*_1/2_ was proved to be high by validation methods. Therefore, a better mutant can be screened in protein engineering. The approach of ISAR in protein engineering could make an effective prediction even if the training set was limited in size, which greatly reduced the cost of the experiment.

In the directed evolution of enzymes, the addition of the activity of a single variant that represented a characteristic was commonly used in many biological methods. Therefore, the inaccuracy of protein engineering for computer processing was probably caused by nonadditive or characteristic variants. The epistatic effect may diminish the experimental effect of protein engineering and the prediction in screening, which was a drawback of previous protein engineering [45–47]. The effect of a combined mutation can be the result of a certain mutation interaction, where the obtained activity value represented the sum of a single activity value, either the activity increase value was larger than that of addition or the activity decrease value was smaller than that of addition. For example, *T*_1/2_ values of 19.19 and 7.35 are obtained for P1 and P2 among the six-point mutants from Table 2. Therefore, it can be found that P1 (I77L) is superior to P2 (Q97E) in physical and chemical properties. However, the *T*_1/2_ values of P7 (I77L_H210N) and P8 (Q97E_H210N) are, respectively, 42.74 and 32.85. As aforementioned, it was not practical to use simple additive activities to predict activities. Every changing point influenced the whole spectrum and activity. In protein engineering, it is crucial for amino acid sequences to have epistatic effects.

## 4. Conclusion

In this study, the ISAR method was based on the binary coding of WT amino acid sequences and the half-life of a group of combinatorial mutants with known thermostability. The statistical model of ISAR was composed of amino acid sequence information and biochemical characteristics of protease expression mutants. The optimal AAindex is determined by several parameters in the AAindex database using the first stage (coding stage) and the fast Fourier transform. Meanwhile, the frequency of the protein spectrum can be changed by Fourier transform, and its amplitude was the transformation of protein properties, independent of the position of each amino acid in the protein sequence. These points interacted with each other to form different protein spectra. Then, the best model was built with better parameters (*R*^2^ and cvRMSE) and LOCCV using limited experimental data and protein spectrum energy. According to the model, the *T*_1/2_ values of 64 possible mutants were predicted and validated. This statistical prediction model was related to the physical and chemical properties of amino acids and amino acid sequences and used the effective conversion method of DSP so that better mutants were obtained.

The biggest advantage of ISAR is that predictions can be made with a small amount of experimental data, and it requires only the amino acid sequence of biological macromolecules. Only the protein variants and their biological and biochemical activity values are needed as the initial dataset to generate the prediction model. Therefore, proteins without spatial structure were also suitable for ISAR. The method effectively alleviated the bottleneck of finding good mutants in the data mutant library. In future research, this method will be used to screen out more excellent mutants of protein enzymes to benefit human beings.

## Figures and Tables

**Table 1:** Transaminase: the top 10 best indexes according to the cross-validation root mean square error.

Index	*R* ^2^	*E*	Data description
NAGK730101	0.8929	4.89	Normalized frequency of alpha-helix (Nagano, 1973)
PALJ810116	0.8919	4.92	Normalized frequency of turn in alpha/beta class (Palau et al., 1981)
TANS770103	0.8239	6.65	Normalized frequency of extended structure (Tanaka-Scheraga, 1977)
GEOR030102	0.7691	6.85	Linker propensity from a 1-linker dataset (George-Heringa, 2003)
RACS770101	0.6603	7.52	Average reduced distance for C-alpha (Rackovsky-Scheraga, 1977)
RACS820107	0.6597	7.53	Average relative fractional occurrence in A0(i-1) (Rackovsky-Scheraga, 1982)
RICJ880111	0.6532	7.53	Relative preference value at C4 (Richardson-Richardson, 1988)
BEGF750102	0.6585	7.53	Conformational parameter of beta-structure (Beghin-Dirkx, 1975)
TANS770110	0.6581	7.54	Normalized frequency of chain reversal (Tanaka-Scheraga, 1977)
CHOP780101	0.6518	7.54	Normalized frequency of beta-turn (Chou-Fasman, 1978a)

**Table 2:** The best model according to different training sets selected by ISAR and the comparison of *T*_1/2_ predicted values of 13 mutants with the experimental results [33].

Variant	Mutations	Predicted *T*_1/2_ (min)	Experimental *T*_1/2_ (min)
WT		7.35	6.9 ± 0.6
P1	I77L	19.19	20.1 ± 0.6
P2	Q97E	7.35	16.5 ± 0.6
P3	H210N	22.38	23.1 ± 0.9
P4	N245D	17.07	14.8 ± 0.6
P5	G292D	13.95	14.8 ± 0.8
P6	I295V	9.15	9.3 ± 0.5
P7	I77L_H210N	42.74	42.2 ± 0.8
P8	Q97E_H210N	32.85	30.6 ± 0.5
P9	H210N_N245D	16.10	18.4 ± 0.6
P10	H210N_G292D	33.88	33.6 ± 0.5
P11	I77L_Q97E_H210N	28.25	31 ± 0.7
P12	I77L_H210N_G292D	30.92	16.7 ± 0.6

**Table 3:** The prediction of the remaining 6 mutants' *T*_1/2_.

Variant	Mutations	Predicted *T*_1/2_
P13	Q97E_H210N_G292D_I295V	57.81
P14	I77L_H210N_N245D	52.37
P15	I77L_H210N_N245D_I295V	51.13
P16	H210N_G292D_I295V	46.67
P17	Q97E_H210N_G292D	45.79
P18	I77L_Q97E_H210N_G292D_I295V	45.62

## Data Availability

Data is available in the manuscript.
